# Boosting a Low-Cost Smart Home Environment with Usage and Access Control Rules

**DOI:** 10.3390/s18061886

**Published:** 2018-06-08

**Authors:** Paolo Barsocchi, Antonello Calabrò, Erina Ferro, Claudio Gennaro, Eda Marchetti, Claudio Vairo

**Affiliations:** Institute of Information Science and Technologies of CNR (CNR-ISTI)-Italy, 56124 Pisa, Italy ; antonello.calabro@isti.cnr.it (A.C.); erina.ferro@isti.cnr.it (E.F.); claudio.gennaro@isti.cnr.it (C.G.); eda.marchetti@isti.cnr.it (E.M.); claudio.vairo@isti.cnr.it (C.V.)

**Keywords:** smart environment, wireless sensor networks, monitoring, machine learning, complex event processing

## Abstract

Smart Home has gained widespread attention due to its flexible integration into everyday life. Pervasive sensing technologies are used to recognize and track the activities that people perform during the day, and to allow communication and cooperation of physical objects. Usually, the available infrastructures and applications leveraging these smart environments have a critical impact on the overall cost of the Smart Home construction, require to be preferably installed during the home construction and are still not user-centric. In this paper, we propose a low cost, easy to install, user-friendly, dynamic and flexible infrastructure able to perform runtime resources management by decoupling the different levels of control rules. The basic idea relies on the usage of off-the-shelf sensors and technologies to guarantee the regular exchange of critical information, without the necessity from the user to develop accurate models for managing resources or regulating their access/usage. This allows us to simplify the continuous updating and improvement, to reduce the maintenance effort and to improve residents’ living and security. A first validation of the proposed infrastructure on a case study is also presented.

## 1. Introduction

A Smart Environment (SE) depends on the communication and cooperation among numerous devices, sensor networks embedded in the environment itself, servers in a fixed infrastructure and mobile devices carried by people. All these devices integrate computation, networking, and physical processes and are able to monitor and control physical objects providing an extremely efficient and economic mean for improving the quality of life and the security of people. However, to make “smart” an environment, it is necessary that the data collected by all these devices are analyzed and interpreted following rules that allow intelligent and prompt decisions, especially when a smart environment has to automatically respond to possible intrusions that can jeopardize the safety of people or the security of objects [1]. By using the information collected by all these sensors, specialized software, i.e., intelligent agents, can reason about the environment and trigger actions to change the state of the environment by means of actuators. The sensor/actuator networks must respond to interoperability, privacy, security, heterogeneity and pro-activity issues; their design implies the use of a middleware for their inter-communicability. Besides the monitoring and actuation features, another important aspect is the possibility of these smart environments to communicate with humans in an easy way [2].

One possible categorization of SEs is the one by Poslad [3], who differentiated smart environments on the basis of systems, services, and devices: virtual (or distributed) computing environments, physical environments, and human environments, or a hybrid combination of these. Virtual computing environments enable smart devices to access pertinent services anywhere and anytime; physical environments may be embedded with a variety of smart devices of different types; human environments, where humans, either individually or collectively, inherently form a smart environment for devices, if humans themselves are equipped with smart tools.

A Smart Home (SH) is an example of a smart environment. Of course, there are many answers to the question what is a “Smart Home”, depending on who is asked. In this paper, we consider a Smart Home as an example of a combination of the three categories before mentioned: a home that is equipped with network-connected products (connected via Wi-Fi, Bluetooth, Konnex, or similar protocols) for controlling, automating and optimizing functions either remotely (by a phone, tablet, or computer) or by means of a dedicated system within the home itself; a home that is able to learn the habits of its inhabitants and to prevent some needs; and a home where security and control systems offer added security when integrated with other intelligent systems present in the home.

Recent proposals for the Smart Home management [4] usually focus on maintenance actions, such as reducing heat and turning off lights and appliances, and on management of different home devices. However, the exigency of having a user-shaped, low-cost and integrated home environment is moving research and industry on the development of home automation systems where sensors are used to gather real-live style about security, safety, and energy saving issues [5]. In this paper, we would like to go a step ahead and to provide a holistic approach for the integration of the most innovative, low-cost, and efficient solutions into the Smart Home environment taking into account: (i) the user-friendly management; (ii) the low impact and cost in the physical architectural integration; and (iii) the maximization of data processing issues and networking and interoperability features [6]. In particular, we would like to leverage the Smart Home for modelling and controlling specific users’ behavior to improve the quality of life and the timely and reliable (critical) information exchange.

Our Smart Home is equipped with many types of sensors, with different technologies: presence sensors, smart cameras for the face recognition, energy consumption sensors, temperature, humidity, and so on. The target is to overcome the needs of using sophisticated sensor nodes or high-performance computation engines. Indeed, the plurality of network-connected smart devices generates a huge amount of data that require to be collected, cross-referred, and interpreted by applying specialized rules in such a way to make decisions and related actions. The challenge of our proposal is to exploit low-cost heterogeneous sensor devices for data collection and to develop the specialized software, usually called Complex Event Processing (CEP), able to analyze and correlate the different heterogeneous sources of information, using the computation facilities commonly available in most of the houses.

Indeed, considering the control point of view, a Smart Home usually involves three levels of rules: (i) rules for managing and correlating sensors data and technologies; (ii) rules able to guide the Smart Home processes, to define the users and the system behaviors, and to protect against possible problems and inconvenience faults; and (iii) rules that manage the access to the building, specific rooms, tools or data to protect against possible malicious use or security flaws (access control rules). However, independently of the formalism adopted, writing such kinds of rules is a hard, verbose, and error-prone activity [7]. Therefore, in some smart environments, the common attitude is to define just the basic control rules that may remain unchanged for a considerable amount of time. Thus, existing solutions generally try to adopt a static specification of the different rules and to centralize their control inside the architecture. As a side effect, the management of dynamic environments, such in the Smart Home case, could become quickly outdated over time, leading to inconsistencies with the different behaviors, technological facilities, and security rules.

Based on previous considerations, the solution proposed in this paper relies on decoupling the different levels of control rules to maximize their effectiveness and to reduce as much as possible their maintenance and updating effort. The control levels considered are the Sensors Rules, which define the sensors behavior and activities; the Usage Control Rules, which define the users and sensors interactions; and the Access Control Rules, which manage the accesses to the different resources expressed through a specific control policy formalism.

The purpose is to perform the continuous control and assessment of the Smart Home environment to improve the quality of life, safety and security of the people living, working, and visiting this context.

To better explain the solution adopted, we describe here a very simple example of Smart Home management, where different kinds of people (parents, children, households and so on) can access a specific area (or rooms) of the building, thus requiring different Sensors, Usage, and Access control rules.

For instance, in the Smart Home, some of the rules that manage and coordinate the sensors (temperature, humidity, CO${}_{2}$, and gas) and the actuators (vaporizer, dehumidifier, heating, and air-conditioner) could be defined considering the specific situation, as in the following: (i) if a child and no adult is in the room, the gas valve is locked off; (ii) if level of CO${}_{2}$ exceeds the boundary range and at least one adult is in the room, a warning message is sent to the people in the room to open the windows; and (iii) when nobody is home, all doors and windows must be closed or a warning message is sent to the apartment’s owner.

These simple rules can be defined once for each specific home and may vary only in the boundary ranges required for the different experimentations, or in case new sensors and/or actuators are installed in the room. Therefore, due to the low variability, the rules can be directly embedded into the engine controlling the sensors’ behavior and sporadically assessed for reducing the overall effort for their maintenance.

However, the above mentioned rules could not completely satisfy the management of the Smart Home and the exigencies of the family. For instance, considering the frequent and common problem of children babysitting, the profiling of the different people in charge of the children control (grandparents, babysitters, family friends and so on) could involve different Sensors, Usage and Access rules depending on the day, time and person involved. An example could be: (i) access to the garage is inhibited to anyone if a babysitter is in the house; (ii) terrace door cannot be opened when grandparents are in the house; (iii) the temperature in the house should not be lower than a certain value in presence of grandparents; and (iv) children access to terrace and garage is inhibited if a family friend is in the house.

These are just simple examples of rules, voluntarily ignoring complex, safe, and security aspects of the Smart Home management. Not all the mentioned rules have to be verified at the same time and in all situations. The peculiarity of the proposed system is to leave the freedom to the SH owners to define each time the more suitable rules for their purposes. This can be done either by specifying the proper rules (through an easy-to-use interface) or selecting the most suitable one from a pre-defined collection of frequently adopted rules. A dedicated user-friendly engine will manage the frequent updates/modifications of the rules behavior, overriding when necessary the previous rules, without an impact on the overall management of the SH.

Of course, the rules specifying the SH management could have been implemented once for all writing a standard usage control policy enforced by a standard usage control engine, as in many current available proposals. One of the challenges of the proposed infrastructure is the possibility of the personalization and customization of the different sets of rules. Specific internal features and tools are in charge of the management of possible errors and inconsistencies to promptly correct them [8,9].

The proposed infrastructure has been validated by installing the selected wireless sensor networks and some smart cameras for surveillance in several rooms, having in mind that the entire proposed infrastructure has to be applicable to any Smart Home environment. The rest of the paper is organized as follows: Section 2 describes the integrated devices used to monitor the Smart Home environment and details the infrastructure used to manage Sensors, Usage and Access control rules by inferring patterns of interest from data coming from the integrated devices. The case study is presented in Section 3, while the overall performance of the proposed infrastructure is shown in Section 4. Section 5 reviews the related work. Finally, Section 6 concludes this paper and suggests future work.

## 2. The Proposed Solution

In this section, an overview of the considered devices is given; in particular, the communication technologies used to monitor the ambient and the technology exploited to identify known persons accessing the environment are described. The information collected from all this technology is able to monitor Sensors, Usage, and Access control rules and to infer patterns of interest from data.

### 2.1. Wireless Sensor Network Devices

In our Smart Home, we consider a wireless sensor network (WSN) composed of sensors with different communication technologies: ZigBee, ZWave, Bluetooth Low Energy (BLE), and Konnex; others technologies can be easily integrated.

ZigBee turns out to be a communication protocol widely used in industrial environments since it supports different network topologies (star, tree, and mesh); it also provides services for network startup, multi-hop routing, and connection/disconnection management of the nodes. The ZigBee choice is driven by several technology characteristics, such as ultra-low power consumption, use of unlicensed radio bands, cheap and easy installation, flexible and extendable networks, integrated intelligence for network set-up and message routing.

ZWave appears to be the emerging wireless technology as regards the domotic scenario because, operating at low frequencies (868–900 MHz), it is not very affected by signal propagation interference. The ZWave technology has been chosen for the gateway that collects all data from sensors. In general, the gateway allows connecting the various ZWave devices to collect data from the sensors thus modifying the physical state of the environment through the use of actuators.

The Bluetooth Low Energy (BLE) protocol, included in the latest version 4.0 of the Bluetooth specifications, is focused on low-power sensors. Although it uses the same frequency band as the classic Bluetooth protocol (the 2.4 GHz ISM band with a free license), the modulation is slightly different, resulting in greater signal strength. With the same transmission power, BLE allows an increase of about 60% in the communication range. Devices that are powered by a single battery can operate for years, depending on the use, of course. The drawback lies in the reduced maximum data rate, which in classic Bluetooth is 10 times better. Nevertheless, for low transmission rates, as typically in the case of sensors, the BLE protocol is more appropriate.

Konnex (KNX) is a European standard for the office automation. KNX can be used in all possible applications and functions for the control of homes and buildings: lighting; the control of rolling shutters; safety; monitoring of heating, ventilation and air conditioning; water control and alarms; energy management; management of electricity meters and appliances; audio systems; etc.

Each sensor node of a WSN can aggregate multiple transducers, such as humidity, temperature, Passive Infrared (PIR) to identify all the indoor movements of users [10], gas sensor, noise detector and power meter to evaluate the real power consumption of the target environment. Moreover, we also used magnetic contacts, gas valve, and power plug. Each node of a WSN is connected to a sink, which provides IoT connectivity through the IPv6 addressing.

**Figure 1:**
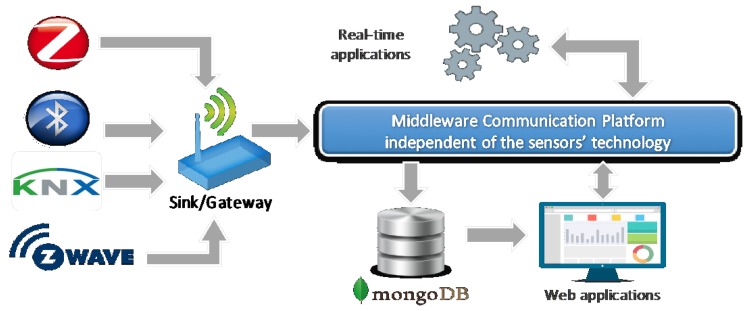
How heterogeneous systems communicate.

The values measured by the wireless sensor network are made available to applications through a middleware platform and data query services [11]. Technically, the middleware is the software layer that allows the communication and management of information in distributed environments. It represents the glue between the high-level applications and the underlying systems. The scheme of our system is represented in Figure 1.

### 2.2. The Smart Home Cameras

**Figure 2:**
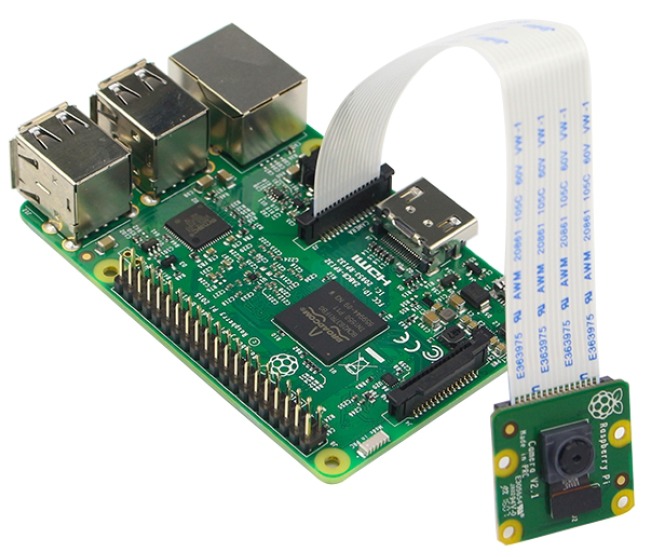
Raspberry Pi 3 with camera module.

The Smart Home Cameras are used for two main purposes: to identify possible intruders (i.e., unknown persons) entering the home and to identify known persons accessing the rooms of the home to make decisions and to personalize services. To achieve these goals, we perform a visual face recognition algorithm by exploiting smart cameras. We refer to a smart camera as a cheap, card-sized single-board computer, equipped with a camera sensor, a memory, and a communication interface, either wired or wireless. In particular, we used the widely adopted Raspberry Pi 3 model B and its standard camera module version 2 as a smart camera (Figure 2), whose total cost is about 80 €. More precisely, the Raspberry Pi 3 hardware includes:

- BCM2837 1.2 GHz Quadcore ARM Cortex-A53, 64 Bit CPU
- 1 GB RAM DDR2
- 32 GB micro SD card for storage

The camera module is an 8 MP fixed-focus camera that supports 1080p30, 720p60 and 480p90 video modes, and image mode at a maximum resolution of 3280 × 2464 pixels.

The system is based on an archive of known persons that is shared among all cameras in the home. This archive contains information that are used at run-time to perform the face recognition task. This information is extracted from ten images of each of the known persons captured during the system setup. It is important to stress that, as we explain below, this archive only contains numerical information about the persons, being the images of the faces not stored in the system.

At run-time, the smart camera autonomously processes the video stream, looks for faces in the captured frames and tries to recognize them. The output of the smart camera processing is the label assigned to the recognized face or a notification of unknown person, if no match with the known persons is found. We used the OpenCV library to process the images acquired by the cameras and to perform the face detection (we used the OpenCV implementation of the Viola–Jones approach [12]) and a Deep Learning approach to determine whether the face seen by the smart camera at run-time belongs to one of the known persons. In particular, we used the VGGFace2 Convolutional Neural Network (CNN) to process the input image and to extract some numerical information from this image (we call this information deep feature), which we used to perform a similarity search with the known faces stored in the archive to determine if there is a match. The VGGFace2 [13] is a ResNet-50 CNN with Squeeze-and-Excitation blocks (shortly SEResNet50) trained on MS-Celeb-1M [14] dataset and fine-tuned on the VGGFace2 [13] dataset. This network architecture works very well both in terms of efficiency and effectiveness, which are very important in an embedding system such as the one proposed in this paper. In fact, the computational time that we experienced with Raspberry Pi 3 hardware to compute the deep feature from an input image is about 3.5 s on SEResNet50, which is acceptable for a non-hard real-time scenario as the one we propose.

As remarked above, the deep features extracted from the faces of the known persons are the only information stored by the Smart Home Cameras; this information is a float vector, and the image itself is discarded after the processing needed to compute the vector. At run-time, when a face is detected, the deep feature for that face is extracted and the image is discarded as well. The deep features stored in the known persons archive are bound to a label identifying the person. This label is set during the system configuration, when the deep features are extracted, and it is not required to be the real person identity.

The interaction with the central server of the Smart Home is required only when a new person has to be added to the known persons’ archive (to download the corresponding information locally to the smart camera) and when a recognition is performed. In this case, the transmitted information is just the label of the recognized face, bound with a confidence, or an unknown person notification, if no match is found. No image or video stream is transmitted outside the smart camera.

### 2.3. The Smart Home Monitoring Network

In this Section, we provide some details about the infrastructure used for Sensors, Usage, and Access control rules and for inferring patterns of interest from data flowing through the sensors and cameras network described in Section 2.1 and Section 2.2. The main goal of this architecture is to decouple the high-level specification of properties related to the behavior, i.e., Sensors, Usage Control and Access control rules, from the events to the physical structure of the sensors.

**Figure 3:**
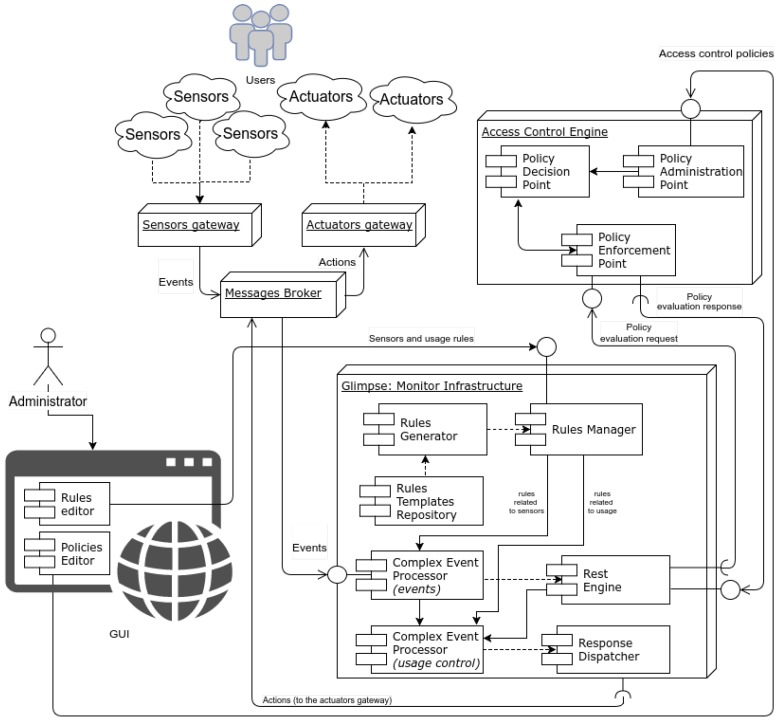
Proposed architecture.

In Figure 3, an overall overview of the deployed infrastructure is shown. The Infrastructure is conceptually divided into different nodes:

- The *Access Control Engine* is the node in charge of implementing the access control management.
- The *Glimpse: Monitoring Infrastructure* is the node monitoring and enforcing the Sensor and Usage rules.
- The *Sensors and Actuators* are physical (hardware) components of the infrastructure (see Section 2.1 and Section 2.2).
- The *Management Interface* is the GUI through which the different rules can be defined and feedbacks and log analysis can be provided.

The Administrators, i.e., the Smart Home owners, are in charge of providing the definition of the three levels of rules for the overall infrastructure. This can be done by means of a user-friendly *GUI*, which *Rules editor* and *Policies editor* components are running on. Specifically, through the *Rules Editor*, the Administrators can define the Sensor and Usage rules, which will be automatically translated into a specific language (further details are provided in Section 2.3.2). Additionally, by means of the *Policy Editor*, the Administrators can define the access control policies, automatically translated into XACML language, that will rule the resources access. Finally, through the *GUI*, the Administrators can visualize logging data, monitoring results, sensors, and actuators status. The definition of the Sensors, Usage, and Access control rules may cause inconsistency and security problems, especially in the case of non-expert users. To mitigate this risk, an automatic facility to specify and formally verify the rules integrity has been included in the provided GUI. In particular considering the access control aspects, it integrates functionalities, based on an attribute-based specification language, derived from the FACPL language [15], that enables both the testing and the automated verification of properties regarding the authorizations enforced by single policies and the relationships among multiple policies. For usage and sensors rules expressed through JBoss Drools, the component integrates the Drools Verifier (JBoss Drools Verifier, https://developer.jboss.org/wiki/DroolsVerifier), a Drools facility dedicated to the rule consistency. Further details can be found in [16,17,18,19].

In the following subsections, more details about the above mentioned nodes are provided.

#### 2.3.1. Access Control Engine

This node manages the resource access by enforcing the XACML Policy defined by the Administrators. In particular, the *Access Control Engine* node contains three components (Figure 3, top right):

- The *Policy Enforcement Point (PEP)*, usually embedded into an application system, receives the access request in its native format from the *Glimpse: Monitoring Infrastructure*, constructs an XACML request and sends it to the *Policy Decision Point (PDP)*. It receives the *PDP* responses and forwards them to the *Glimpse: Monitoring Infrastructure* through its *REST Engine Interface*.
- The *Policy Decision Point (PDP)* evaluates the policy with respect to the request and returns the response, including the authorization decision to the *PEP*.
- The *Policy Administration Point (PAP)* is the component entity in charge of managing the policies and deploying them on the *PDP*; it receives the XACML access control policy by the *Management Interface*.

#### 2.3.2. Glimpse: Monitoring Infrastructure

The monitor infrastructure is integrated into the proposed infrastructure called *GLIMPSE*; it is a flexible, adaptable and dynamic solution independent of any specific sensor or access control network notation or execution. It provides a monitoring infrastructure able to perform complex event recognition, as well as observing and notifying timely specific event occurrences.

*GLIMPSE*, developed using Java technologies, is a Maven project available on GitHub (*GLIMPSE*, https://github.com/acalabro/glimpse). The infrastructure is modular and composed of several components, that can be executed accordingly to the usage needed.

*GLIMPSE* allows us to take countermeasures for recovering from violations of defined performance constraints. These constraints are not mandatorily specified at the system startup, but can be automatically raised by the rule engines involved or can be improved at runtime by injecting new rules on the complex event processors. This makes the system able to proactively select suitable mechanisms for guaranteeing self-recovery, self-configuration, and self-repairing of the application or System under Test (SuT) [20]. The monitoring framework presented in this paper has been inspired by the monitoring architecture presented in [21,22].

**Figure 4:**
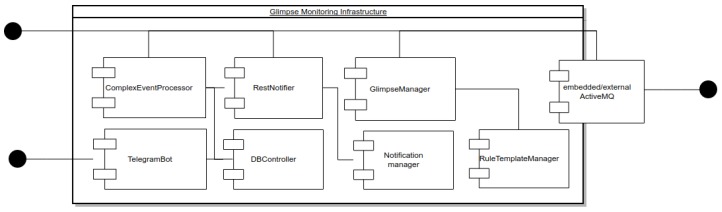
Glimpse monitoring infrastructure overview.

A general architecture description is provided in Figure 4.

The *Glimpse Monitoring Infrastructure* node (Figure 3) manages the complex event processing and the interactions with *Sensors*, *Actuators* and *Access Control Engine*, and includes new features devoted to the usage and access control request generation.

The main monitoring components are:

- The *Rules Manager* component is in charge of orchestrating the rules generation starting from the templates stored within the component *Rule templates Repository* through the *Rules Generator* component.
- The *Rules Generator* is the component in charge of synthesizing the rules starting from the directives received by the *Rules Manager* by means of techniques based on generative programming approaches [23,24].
- The *Rules Templates Manager* is an additional internal repository storing the meta-rules enabling the run-time adaptation by means of generative procedures.
- The *CEP-Events* is a rule engine realized by means of the Drools rule language (Drools Fusion: Complex Event Processor, http://www.jboss.org/drools/drools-fusion.html). It correlates the events flowing from *Sensors* with the rules loaded by the *Rules Manager* component.
- The *CEP-Usage* is in charge of correlating complex events generated by the *CEP-Events* with the rules related to the usage of the resources, loaded by the *Rules Manager*.
- The *Rest Engine* is the component in charge of communicating through REST [25] interfaces with the *Access Control Engine* to send/receive the *Access Control Engine* request/response.
- The *Response Dispatcher* through the *Message Broker (AMQ)* sends events to the actuators managed by the *Actuators gateway*.

The peculiarity of the proposed architecture is to include a chain of two CEP entities, the *CEP-Events* and the *CEP-Usage*, for decoupling the activities concerning the management of the sensors from those more related to the administration of the resource usage and alarming situations. This makes easier the definition of new primitive events generated by (new/updated) sensors and the inferring of events in the form of composite events in a way completely independent of the access and usage control rules. Moreover, it lets a quick and high-level updating of the general resource access and usage regulations and the planning of specific corrective actions in the case of alarms or resource violations, leveraging from the specific sensor network on which they are implemented.

**Figure 5:**
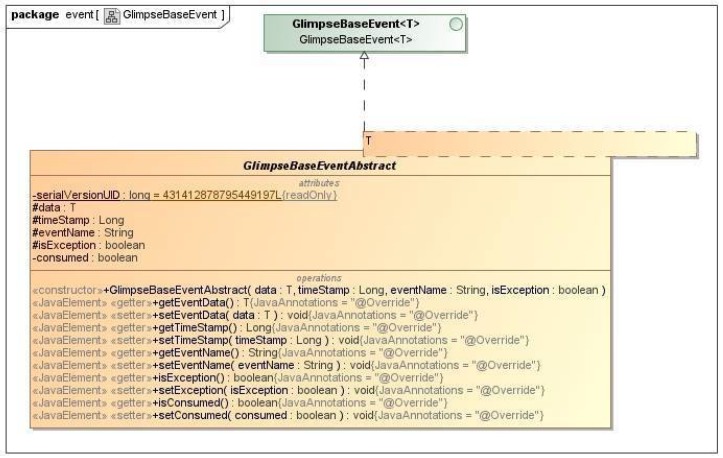
Event description.

From a practical point of view, all communications among monitoring components are performed through [26] messages sent to the *Message Broker*, also known as *AMQ*. An embedded version is executed if the external one is not available. For improving the security of the communications, *GLIMPSE* is able to adopt Secure Sockets Layer (SSL) connections with mutual authentication with self-signed certificates among consumers, probes, and brokers. Events managed by *GLIMPSE* are structured as shown in Figure 5.

## 3. Case Study

In this Section, we describe the usage of the proposed infrastructure for the management of a hypothetic Smart Home (SH). Here, we report just a simplified description of the management of the Smart Home as our main goal is not to enter in details of complex rules but rather to underline the role and the advantages of the proposed infrastructure.

The SH focuses on three different main aspects: to keep the values of sensors (temperature, humidity, CO${}_{2}$ and gas) under a safe and secure range; to rule the access to the SH and to the different rooms of the building; and to activate corrective actions in the case of detected violations or alarming situations. For these purposes, the room has been instrumented with different sensors, and several sets of control rules have been defined. These last include: (i) Sensor Rules for managing the security boundary value of each sensor or a combination of them (for instance, the tolerance temperature, humidity ranges, and so on); (ii) Access Control policies ruling who and when can access the SH or a specific room (name and role of people allowed to access); and (iii) Usage Control Rules for managing user profiles, sensors failures, resource violations, and alarming situations in general (for instance, if a child and no adults are in the room, the gas valve is locked off).

### 3.1. Case Study Set Up

**Figure 6:**
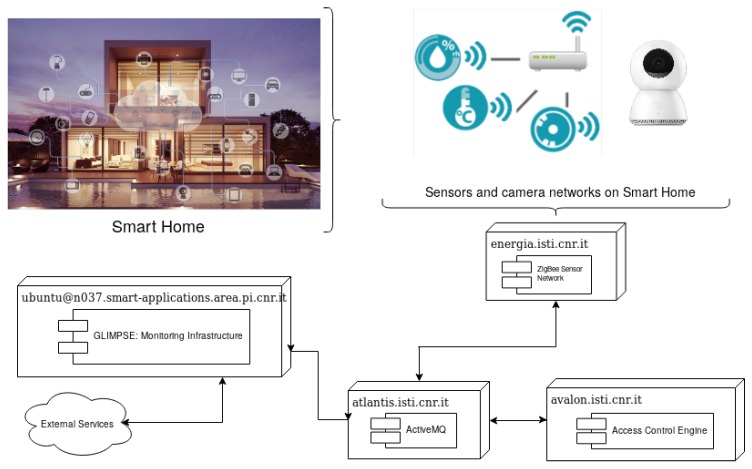
Deployment configuration.

As shown in Figure 6, the SH rooms have been equipped with several sensors (see Section 2.1 for more details): temperature and humidity, presence, power meter, noise detector, gas sensor, magnetic contact, gas valve, and power plug. All these devices have been deployed in 10 office rooms emulating a real house installation. We also installed two smart cameras in two offices in order to emulate the monitoring of the entrance room and of the kitchen of the SH. The total number of devices deployed is about 50 sensor devices and 2 smart cameras, in an area of about 200 square meters. The proposed system has been validated through a measurement campaign of six months, where each sensor’s data have been stored in the SH database, together with the data produced by the smart cameras.

### 3.2. Sensors, Access and Usage Rules Management

In this Section, we focus on the interaction among *CEP-Events*, *CEP-Usage*, and the *Access Control Engine* for the enforcement of the sensors, access, and usage rules. Specifically, as shown in Figure 3, through the *Rules Editor* the Administrators loaded the sensor rules useful for monitoring the sensors status and the access rules for the identification of who is currently asking resource access.

When an owner or an authorized guest (grandparents, babysitter and so on), through the face recognition system, is identified, the *CEP-Events* receives the access request and extracts the room ID and the identity of the person (person ID). By querying the SH database, the *CEP-Events* retrieves (Role, allowed room ids) attributes related to the user who is asking for the access. Using the data collected, the *CEP-Events* sends a *Policy evaluation request* through the *Rest Engine* to the *Access Control Engine* node and a *PdpAccessRequest* event to the *CEP-Usage* to notify that an access request has been sent. The *PEP* translates the request into an XACML access request and sends it to the *PDP*, which evaluates the request according to the access control policies injected by *Policy Administration Point (PAP)*, and sends back the response to the *PEP*, which in turn sends back to the *CEP-Usage* through the *Rest Engine*.

An example of a sensor rule used by the *CEP-Events* for controlling all the installed sensors is shown in Listing 1. In particular, when the *CEP-Events* receives from the monitored sensors, for two consecutive times, null or out-of-range values (lines 13–14 and 17–18 of Listing 1), the *CEP-Events* generates a complex event called *SensorFailureEvent* for notifying the detected failure to the *CEP-Usage*, so that it can activate the corrective actions. For aim of simplicity, we do not provide here the formal specification of the XACML access control policies adopted for managing access to the different rooms of the SH. We just report some of the rules implemented in SH access control policies to better explain the potentialities and features of the proposed infrastructure. Among the different types of rooms (resources) of the SH access control policies, here we focus on three of them: living room, garage, and kitchen.

The SH access control policies specify different kinds of users (subjects) such as adults and children. The policies also manage several types of actions for each room: for instance, the access to the garage is inhibited if the babysitter is in the house.


**Listing 1: Usage Control Rules**


[..setup and import omitted..]
			  
declare SensorFailureEvent
 @idroom: int
 @idsensor: int
end
  			  
rule “Check data from temperature sensor”
  no-loop true
  salience 1
  dialect “java”
  			  
when
  $aEvent:GlimpseBaseEventSB(this.isConsumed == false,
  this.isException == false,(this.getTemperature == null || < −20 || > 0 ) );
  $bEvent:GlimpseBaseEventSB(this.isConsumed == false,
  this.isException == false,
  (this.getTemperature == null || < −20 || > 0 ),
  this after $aEvent,this.getSensorID == $aEvent.getSensorID);
then
  SensorFailureEvent failureDetected = new SensorFailureEvent(idRoom,idSensor);
  CepBinder.sendEventTo(“CEP - Usage”, failureDetected);
  $aEvent.setConsumed(true);$bEvent.setConsumed(true);
  retract($aEvent);retract($bEvent);
end  
			  


Finally, the access control policies specify different environment values and conditions; for the sake of simplicity, here, we only consider the case in which the environment represents the different time slots in which a person can access the different rooms. For instance:

1. ARule 1: The babysitter can access the SH in the afternoon (from 1:00 p.m. to 8:00 p.m.).
2. ARule 2: The grandparents can only access the garage during the day.
3. ARule 3: The owners can access the garage at any time.

At run-time, the request sent by the *CEP-Events* to the *Access Control Engine* is evaluated by the *PDP* component and the corresponding reply is sent back to the *CEP-Usage*, which uses the received (permit or deny) response to allow the resource access or to deny it in case of possible violations or resource misuses. In both cases, the *CEP-Usage* is in charge of notifying the Actuators of the (corrective) actions to be executed. For instance, the temperature should be kept at a certain value when grandparents are in the house.

Example of usage rules can be:

1. URule 1: If a child and no adults are in the room, the gas valve is locked off.
2. URUle 2: The terrace door cannot be opened when grandparents are in the house.
3. URule 3: If the level of CO${}_{2}$ exceeds the boundary range and at least one adult is in the room, a warning message is sent to the people in the room to open the windows.
4. URule 4: When nobody is in the house, all doors and windows must be closed. If not, a warning message is sent to the apartment’s owner.
5. URule 5: When gas sensor notifies that the value is out of safe range, the main gas valve is immediately closed and a warning message is sent to the apartment’s owner.
6. URule 6: SH (or specific room) access is inhibited if there are ongoing alarms compromising the safety or security of the guests.

In particular, considering for instance URule 6, a specific instantiation in case of gas alarm is shown in Listing 2. Indeed, the rule checks if there are pending access requests to the SH from any kind of users and ongoing alarms coming from the gas sensors (activation of URule 4) not already solved by the SH owners (for instance, forcing the windows opening). In this case, the rule : (i) forces the inhibition of any possible access to SH, apart from the SH owners; and (ii) retrieves from the SH database the contacts data of the users trying to access (if there are any) and sends a message of SH access deny.

Here, we describe the management of the scenario in which an alarm is raised by the sensors (gas alarm) and the relevant corrective actions are activated by the SH owners. The scenario preconditions are the following: (i) each frequent guest (babysitter, grandparents, and family friends) is registered on the internal SH personal database; (ii) each authorized person accessing the different rooms can be identified through the face recognition system; (iii) each room is constantly monitored by sensors able to send events to the Monitoring Infrastructure; and (iv) no one is currently inside the kitchen and sensor values are within their allowed ranges.

Initially, an adult accesses the house and, according to the interaction described in Section 3.2, an event is sent to the *CEP-Events* through the *Message Broker* and the proper access request is sent to the *Access Control Engine*. This last evaluates the request and sends back the response to the *PEP*, which in turn sends back to the *CEP-Usage* through the *Rest Engine*.

As shown in Listing 2, if any revocation of permission is ongoing (line 8), there are no critical conditions (i.e., the values of temperature, humidity, gas, energy consumption, and noise are in the allowed ranges-line 16), and *PDP* response includes a permit (i.e., the adult recognized as authorized to access the house during the allowed time slot-line 15), the *CEP-Usage* sends an event to the *Actuator gateway* for enabling the door opening through the *Response Dispatcher*.

Supposing, instead, that a critical condition has been detected by *CEP-Events* (for instance, the gas value is out of range or there are significant variations in the noise or in the temperature), a *SensorFailureEvent* event is sent to the *CEP-Usage* (line 19 of Listing 1). This last overrides the *PDP* response, thus allowing adults to access, and sends an event to the *Actuator gateway* for enabling the door opening to the SH owners only (line 45–46 of Listing 2).

Moreover, the *CEP-Usage* sends an alarm event to the SH owners through a specific *Actuator gateway* for requesting authorization of exceptional corrective actions such, for instance, “open the kitchen windows”.


**Listing 2: Access Control and Safety/Security Rules**


[..setup and import omitted..]
  
declare SensorFailureEvent
 @idroom: int
 @idsensor: int
end
   
rule “If there are NOT pending alarm forward PDP access response”
no-loop true
salience 1
dialect “java”
   
when
  $aEvent:PdpAccessRequest();
  $bEvent:PdpAccessResponse(this.isConsumed == false,
  this.isException == false,
  this.getIdRequest == $aEvent.getIdRequest,
  ($bEvent.getResponse == “Permit” || “Deny”),this after $aEvent);
  not(SensorFailureEvent(this.isConsumed == false,
  this.isException == false,this.idRoom == $aEvent.idRoom,
  this.idSensor == $aEvent.idSensor));
then
  Actuators.ManageAccess($aEvent.getIdSensor(),
  $aEvent.getIdroom(),$bEvent.getResponse()));
end
                    
rule “If there are failures take countermeasures”
no-loop true
salience 1
dialect “java”
            
when
  $aEvent:SensorFailureEvent();
then
  Alarm.NotifyToOwners($aEvent.idsensor, $aEvent.idroom);
  //or take direct countermeasure through actuator
end
         
rule “If there are pending alarm check accesses to the resource”
no-loop true
salience 1
dialect “java”
   
when
  $aEvent:PdpAccessRequest();
  $bEvent:PdpAccessResponse(this.isConsumed == false,
  this.isException == false,
  this.getIdRequest == $aEvent.getIdRequest,
  ($bEvent.getResponse == “Permit” || “Deny”),this after $aEvent);
  $cEvent:SensorFailureEvent(this.isConsumed == false,
  this.isException == false,this.idRoom == $aEvent.idRoom);
then
  Actuators.ManageAccess($aEvent.getIdSensor(),$aEvent.getIdroom(),
  OwnersDatabase.NotificationToParentsCheck($aEvent.getIdUser));
end
			  


## 4. Validation Analysis

To validate the proposed system, we evaluated the performance of both the face recognition system and the entire Smart Home monitoring infrastructure. The performance of the face recognition has been measured in terms of accuracy, false positive and false negative rate, while the proposed SH monitoring system in terms of robustness and reliability.

### 4.1. Face Recognition Validation

In this section, we describe some experiments performed to assess the accuracy of the system in performing the face recognition task by means of the Smart Home Cameras. To perform these experiments, we created a ground-truth dataset of 14,380 images of 44 recognized people taken in the office rooms were the smart cameras have been installed in the period ranging from April to October 2017. We refer to this number of faces as $N_{R}$. The dataset also contains a set of 1013 images of unknown people (but that we know do not belong to the group of 44 of the aforementioned identities), which is used for testing the ability of our classifier to recognize intruders. We call this number $N_{U}$.

The face recognition task has been executed by exploiting a *k* Nearest Neighbor (*k*NN) algorithm. We used each of the faces detected in the camera video stream as a query for a *k*NN search in a training set composed of ten representative images for each of the 44 authorized persons and we extract the features from them, using the VGGFace2 CNN described in Section 2.2. We used the Euclidean distance as a dissimilarity measure between features and we sorted the entire dataset of 440 features according to this distance with the given query, from the nearest to the farthest, and we took the first *k* items. The result of this operation is the set *K* of labeled faces belonging to the training set, ordered with respect to the increasing values of the distance. The label assigned to query the classifier is the class that minimizes the sum of similarities between the query and the faces labeled in the ranked list *K* [27]. The confidence of the classification is computed as $1-d_{f}$, where $d_{f}$ is the distance of the first face in *K* from the query and can be thought as the probability of the predicted label to be correct. The distances are normalized so that they are always less than or equal to one.

**Figure 7:**
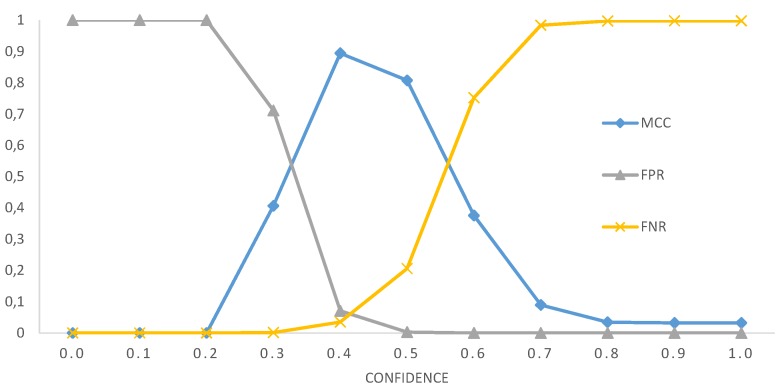
Performance figures of VGG2.

We used a confidence threshold to decide whether or not a person is to be considered as known or unknown during the identification phase. If the probability of the most probable labels is below the low threshold, the person’s identity is claimed as unknown. To find the optimal confidence thresholds, we evaluated the False Positive Rate and the False Negative Rate (FNR).

$$FPR=\frac{FP}{N_{U}}\;\;\;\;\;\;\;FNR=\frac{FN}{N_{R}}.$$

where FP is the number of false positives, i.e., the number of unknown faces that have been wrongly recognized by the system, and FN is the number of False Negatives, i.e, the number of known persons wrongly unrecognized by the system. Figure 7 shows FPR and FNR as functions of the confidence. In order to find the optimal values of the confidence, we evaluate the Matthews correlation coefficient (MCC), which ranges from −1 (when the classification is always in total disagreement with the observation) to 1 (when it is always correct). This correlation is maximum when FPR is equal to FNR, which, in our case, occurs when the confidence is 0.42. For this value of the confidence, we obtain a recognition accuracy of the known persons of 96%.

### 4.2. Smart Home Monitoring Data Analysis

During the six months of simulations, several data have been collected from the devices deployed in the 10 office rooms chosen to emulate a real house installation. In some cases, stressful/anomalous situations have been forced to better evaluate the boundary limits of the proposed infrastructure. In such a period, 18,952,795 events have been observed and more than 15,000 correlations related to activities and behaviors in the different rooms have been derived.

In particular, considering the interactions between monitor infrastructure and smart cameras, 39,193 events relative to the face recognition have been sent to the monitor infrastructure. Among them, 12,254 have been recognized as not authorized or not allowed to access the rooms. In this last cases, the monitoring infrastructure also managed the intrusion notification to the room owners by means of a Telegram Bot support. 9133 telegram requests have been satisfied over the six-month measurement campaign.

**Figure 8:**
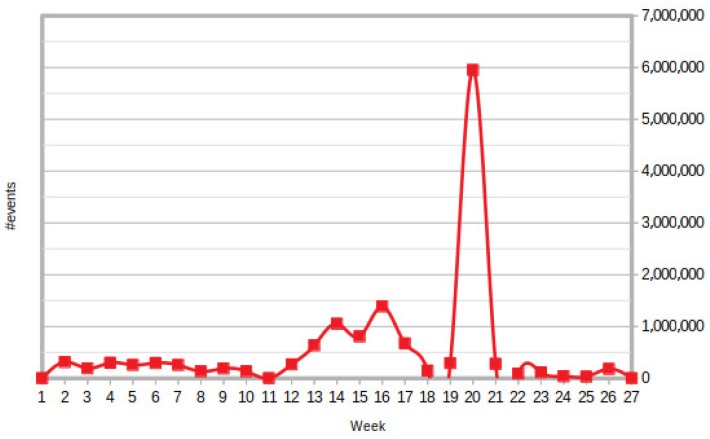
Monitoring infrastructure events flow per week.

To test the robustness of the monitoring infrastructure, a random generation of different kinds of events coming from different sources has been implemented. In particular, we simulated different stress peaks, one of them consisting in the transmission of 3 millions of events in 24 h to the monitoring infrastructure. This last successfully managed this huge amount of events, maintain a good quality of service: no information has been lost and the overall performance values have not been downgraded. For the sake of completeness, we show in Figure 8 the weekly report of the events collected as well as the details of the simulated stress peaks.

Considering, in particular, the resource consumption, the version adopted in the experiment is currently running on a tiny Linux Virtual machine with 4 GB ram and 2 core CPU. This confirms that the proposed infrastructure relies on every accessible resource and allows the leveraging of any smart environment.

## 5. Related Work

This Section reports some works available in the literature that are similar to the presented work in many aspects; however, we mainly focus on differences and strengths on technological capabilities. Our solution is general and is applicable to different scenarios of Smart Home, such as child and elder care, home security, and energy management.

The use of face information to recognize the identity of a person is a research area experiencing rapid development, thanks to recent advances in deep learning. Deep features learned from CNNs have shown impressive performance in classification and recognition problems. For instance, 99.77% ccuracy in the Labeled Faces in the Wild dataset [28] was achieved by Liu at al. [29] and 99.33% by Schroff et al. of Google [30]. To the best of the authors’ knowledge, there are no complete Smart Home systems that use deep learning technologies for face recognition. The Smart Home system proposed by [31] uses unspecified low-level visual features combined with speech features for the sole purpose of recognizing a set of appliance-control commands. The work [32] presents a Smart Home system that includes a face recognition module based on low-level visual features, without performing any experiment to estimate the accuracy of the recognition. Other Smart Home applications that only focus on facial recognition offer raspberry-based embedded systems [33,34,35,36,37]. None of these uses deep learning for facial recognition, and do not even report the accuracy of the recognition, with the exception of [34], which however is limited since it has been carried out only on one authorized person and few unauthorized persons.

One of the increasing applications of the monitoring activity inside the Smart Home environment is the possibility of enhancing elderly living activities. Indeed, the growth and propagation of smart sensors, devices, information, and communication technology let the possibility to exploit the multi-sensory information for establishing Smart Home health monitoring and care. Infrastructures that can automatically control the on/off switching of electronic devices installed in different rooms of a house by speech or video commands are currently challenges solutions [31,33,38,39]. Moreover, in the literature, there are a lot of works that propose a monitoring system designed to save the energy consumed in home environments [40,41,42]. The system we proposed is also able to provide an energy monitoring system but, up to now, we taken into consideration simple rules such as to switch on/off lights and fan coils; however, we plan to investigate and integrate energy saving policies in the future works.

It is out of the scope of this paper to provide a complete survey of the different available proposals dealing these topics; we just mention and compare in the table below those solutions closest to our system. This paper does not go deeply into the details of the health and energy monitoring because the features and facilities included in our proposal could be considered an extension and improvement of the different available solutions for enhancing elderly living activities. Indeed, all sensor, access, and usage rules could be customized to include the management of specific diseases, healthcare conditions, energy saving policies and constraints.

The inclusion of access and usage control in the IoT environments is currently a challenge in many real-world environments [43]. Indeed, the possibility to control mechanisms and devices depending on specific access rules and behaviors is opening the path to the realization of interactive, integrated and customizable environments [44]. In the specific context of Smart Home, the first integrated solutions are the result of the last years of research activity. Among them, here we mention the closest to the proposal of this paper, i.e., the UCIoT (Usage Control in IoT) [45]. This is a fault tolerant and adaptable framework for the enforcement of usage control policies in IoT environments The framework, which integrates a static specification of the different rules and a centralized control, is designed for heterogeneous and distributed architectures of connected devices, evaluating and enforcing security, safety or general purpose policies. The proposal of this paper extends the framework by decoupling the different levels of control rules to maximize their effectiveness and reduce as much as possible their maintenance and updating effort. Among the various practical solutions and patents here we mention one of the most recent, presented in [46]. The proposal integrates different sensors (such as temperature, humidity, accelerometer, microphone optical sensors, and camera), and provides a user-interface to manage the sensors behavior and interaction. The proposal of this paper extends the user-interface with the possibility of specifying integrated sensors, access and usage rules for a better customization of the users’ needs. The RUBICON project [47,48] was dedicated to building robot ecologies consisting of software components, sensors, effectors and mobile robot devices collectively able to deliver adaptive and personalized services thanks to cognitive capabilities such as learning, reasoning and planning. RUBICON learns through an incremental and progressive approach driven by the observation of user’s preference and activities. The learning phase, however, takes time, and often takes many iterations, before the system can adapt to the habits and the preferences of its user.

Finally, considering the use of monitoring engine for access control assessment, several general-purpose monitoring proposals are currently available, which can be mainly divided into two groups: those that are embedded in the execution engine (e.g., [49,50]) and those that can be integrated into the execution framework as an additional component (e.g., [8,51,52]). Both types of solutions have specific advantages. An embedded solution definitely reduces the performance delay of the execution framework, mainly in terms of interactions and communication time. Rules can be directly evaluated by the execution framework, which can also execute corrective actions in case of important deviations. The main disadvantage of these approaches is the lack of flexibility in the data collection. Usually, in these proposals, all the interesting parameters have to be predefined and modeled directly into the execution engine, by means of specific editors. Thus, any change requires redesigning or improving the execution engine itself, thus preventing the possibility of dynamic modifications. The proposal of this paper overcomes the above mentioned issues by proposing a flexible independent monitoring framework able to implement several kinds of rules and usage behaviors.

When the amount of data becomes an issue (such as events from multiple sensors, activity logs, and energy data) platforms can represent a flexible way to store and distribute data more easily and transparently. The Smart Home framework proposed by Hossain et al. [31] exploits the potential of cloud computing and big data technologies to provide load balancing and auto-scaling functionalities that can support a massive number of Smart Home users. The advantages of the cloud come with a cost, the possibility of latencies due to the transmission of information (data and events) to the data center to be processed. In [31], this problem has been addressed by either adding a new instance to support the new load or by saving the data geographically to the regions so that they can be accessed at the fastest speed. As stated in [53], Fog computing can provide real-time streaming processing rather than batch processing to latency-sensitive applications, in which complex event processing is needed. FI-WARE is emerging as a core standard platform for Smart and Connected Communities [54]. The FI-WARE platform is producing new tools to facilitate the development of application and fostering a major inclusion of software standards for smart environments. These tools are provided as software components that can be configured and deployed on a cloud platform in order to easily implement an application. Another important enabling platform is represented by the universAAL architecture, with a particular focus on IoT and Ambient Assisted Living scenarios [55]. Besides its concrete open source implementation, universAAL proposes an architectural reference model based on a set of virtual communication buses as semantic brokers for context events, semantic service requests [56] and user interaction functions. In [57], the authors introduced a framework that sets the basics of a home application server and allows an easy development of home applications.

Our proposal is based on the use of low-cost household equipment that does not use large-scale resources such as the cloud computing services. Table 1 provides a summary of the features of the of the Smart Home Systems previously described.

## 6. Discussion, Conclusions and Future Work

In this paper, we present the technologies, models, and approaches we adopted for developing our low cost, easy to install, user-friendly, dynamic and flexible infrastructure. The main purpose is to demonstrate that it is possible to leverage any environment to a smart one by using basic, off-the-shelf technologies and applications.

The main advantage of the proposed solution relies on the possibility of decoupling the different levels of rules for managing the resources usage and access. Specifically, three levels of rules are defined: the Sensors Rules for correlating sensors data and technologies; the Usage Control rules, which define the users and sensors interactions; and the Access Control Rules, which manage the accesses to the different resources expressed through a specific control policy formalism. A smart interface allows even not-IoT experts to easily maintain and update the different rules and to specify the more suitable corrective actions when changes or constraints violations are notified. Considering the realization of the smart camera on an embedded device, such as a Raspberry Pi, permits a complete decentralization of the computation of the face recognition algorithms, thus facilitating the integration of the system and reducing the bottlenecks. Since typically the number of people to be recognized in a home scenario is the order of tens, this decentralization is feasible simply by replicating the features of people on each smart camera. In the future, therefore, it will be possible to manage a network of smart cameras in a plug-and-play fashion, so that when a new camera is added, the other cameras in the system automatically transmit the archive of the known faces to the new camera.

A first validation on a real case study, considering a simulated Smart Home environment, has been described. The presented scenario evidenced the effectiveness of the proposed approach to correlate events generated by different sensors and to leverage different levels of rules for raising alarms, when critical situations are detected. As a future work, we intend to validate the proposed solution in real-world environments with different peculiarities and security constraints, as well as different rule specification languages. Moreover, we already started running the SH infrastructure on top of a cloud platform and its instances executions actually does not raise any functional issue; we will provide more valuable results in future works. We are also refining the smart interface to make easier the user-interaction and to provide a more effective and efficient automatic derivation of the different monitoring, usage, and access rules. Moreover, we plan to extend the infrastructure to include more refined levels of rules, further decoupling the management and control functionalities of the proposed infrastructure. Finally, we are studying the possibility to include in the infrastructure some online testing facilities to better and continuously guarantee the level of security and privacy required by the recently adopted standards.

## Figures and Tables

**Table 1:** Summary of the features of the of some Smart Home Systems described in the literature.

Features	Smart Home Systems									
	Ours	RUBICON [47]	Ma et al. [32]	Zhang et al. [38]	Hossain et al. [31]	Vikram et al. [58]	Fadell et al. [46]	Escoffier et al. [57]	FI-WARE [54]	UniversAAL [56]
Low-cost	x	x		x			x	x		
Face Recognition	x		x		x					
Interoperable	x	x		x	x	x	x		x	x
Easy-to-use	x	x	x	x	x	x	x	x		
Dynamically adaptable in the nature of sensors	x	x	x			x	x		x	x
Predefined sensor rules	x			x	x	x				x
Customizable sensor rules	x	x					x			x
Customizable usage rules	x	x					x			
Customizable access rules	x	x								
Low computational power	x		x	x		x	x	x		
Cloud-based				x					x	
Privacy preserving	x								x	x
